# Online Formation of Companies in Lithuania in a Comparative Context: Implementation of the Digitalisation Directive and Beyond

**DOI:** 10.1007/s40804-023-00282-6

**Published:** 2023-03-22

**Authors:** Virginijus Bitė, Ivan Romashchenko

**Affiliations:** 1 Professor (Law), Institute of Private Law, School of Law, Mykolas Romeris University, Vilnius, Lithuania; 2 Senior Researcher (Law), Legal Technology (LegalTech) Centre, School of Law, Mykolas Romeris University, Vilnius, Lithuania

**Keywords:** Online formation of companies, Digitalisation Directive, Model constituent document, Electronic signature, Limited liability company, Digital technologies

## Abstract

For many years, paper was the main format for the registration of companies. The Digitalisation Directive, adopted in 2019, obliged European Union (EU) Member States to provide founders with the option to form private companies digitally. Although for Lithuania, where online formation of legal entities had already existed even before 2019, these regulatory developments did not bring about radical change, they forced the national legislator to introduce the required amendments. This article aims at studying the provisions of the Digitalisation Directive and the results of its implementation in Lithuania to suggest possible improvements in the online registration of companies. The laws of both EU jurisdictions (Estonia, Latvia, and Poland) and one non-EU jurisdiction (Ukraine) with experience of online registration of companies are investigated in comparison with Lithuanian law. In addition, the results of a survey Among Lithuanian law firms are presented and contribute to the analysis and interpretation of the legislation at hand. The article’s conclusions about the state of implementation of the Digitalisation Directive and the recommended steps beyond the Directive’s transposition provide for further enhancement of statutes and practices in this area.

## Introduction

Every year one can observe that the role of technologies in modern life increases. The reality of the COVID-19 outbreak intensified the use of digital means and accelerated technological progress in many areas. Companies and corporate governance were no exception. Various digital tools are actively being used both in cases of internal corporate relations, where a company and its shareholders are involved, and in external relations, where a company interacts with the state by filing information and documents, obtaining permits and licenses, asking for approvals, etc. Due to their wide application, digital technologies undoubtedly present a challenge for the regulation of relations where companies, their shareholders and officers are involved.^1^ That is why the instruments of digitalisation that are used in corporate mechanisms need to be carefully studied to suggest possible improvements in this area.

Many legal scholars have already contributed to research about the use of digital tools in company law and corporate governance. Topics that are traditional for company law, including directors’ duties and shareholder empowerment, have gained new meaning due to the use of technologies and risks that have come therewith.^2^ In addition, the increased use of artificial intelligence has forced scholars to ask themselves whether corporate technologies can fully replace corporate boards^3^—a question which was convincingly viewed as wrong by Luca Enriques and Dirk A. Zetzsche.^4^ Moreover, in the debate about the role of artificial intelligence, some researchers, also referred to as ‘tech proponents’,^5^ have viewed digitalisation as an effective way to eradicate existing human-related corporate governance issues.^6^ This optimistic approach requires deeper study, but even without delving into the arguments for believing in the bright future of corporate technologies one may spot the advantages of digitalisation. As Eleanore Hickman and Martin Petrin claimed, artificial intelligence has the potential to bring very positive changes, including quicker processing of large amounts of information.^7^ At the same time, the probable negative effects of new digital technologies, e.g., data and privacy breaches, are largely unknown.^8^ Besides the increased focus of legal scholarship on the implications of technologies in recent years, the development of the EU Company Law has hastened. After the adoption of the general codification Directive 2017/1132 on certain aspects of company law (hereinafter—the Company Law Directive)^9^ more initiatives followed. In 2018, the so-called Company Law Package was published by the European Commission: it included two proposals for directives. One of them was the Proposal for a Directive amending Directive (EU) 2017/1132 regarding the use of digital tools and processes in company law,^10^ which eventually became Directive 2019/1151 (hereinafter—the Digitalisation Directive).^11^ After the proposal on the Digitalisation Directive was submitted, some publications regarding the prospects of adoption of this Directive and its implementation in Germany appeared from, among others, Florian Moslein,^12^ Sebastian Omlor^13^ and Christoph Teichmann.^14^ The challenges the proposal on the Digitalisation Directive brought included the alignment of differing incorporation approaches in Member States and the cross-border issues of company formation.^15^ The second part of the Company Law Package was the Proposal for a Directive amending Directive (EU) 2017/1132 as regards cross-border conversions, mergers and divisions.^16^ Both proposals of the Company Law Package were adopted in 2019 and encouraged the EU Member States to implement new digital practices in their national laws.

This article focuses on the implementation of the Digitalisation Directive as far as company formation is concerned, with a particular focus on Lithuanian law. The purpose of the article is: to assess whether the provisions of the Digitalisation Directive are sufficient to satisfy the declared goals regarding online registration of companies as well as the results (adopted acts and draft documents) of implementation of the Digitalisation Directive already carried out and intended in Lithuania; and to suggest possible improvements in online registration of companies. To a large extent, the article deals with the Directive’s provisions and the respective implementation regulations as far as the first deadline for implementation is concerned: 1 August 2021 or 1 August 2022, if an extension was applied. Of the jurisdictions under investigation, Lithuania, Latvia, and Estonia have not applied for an extension.^17^ Meanwhile, for Poland and some other EU Member States, the first transposition deadline was 1 August 2022.^18^

The article primarily focuses on Lithuanian law and experience in implementing the Digitalisation Directive and some historical aspects of its preparation. Besides the Lithuanian legal statutes and practice, the law of Poland as well as the laws of Latvia and Estonia as EU jurisdictions and the law of Ukraine as a jurisdiction beyond the EU are studied from the comparative perspective. Besides the geographical closeness, common historic routes, and comparable legal systems, all the mentioned jurisdictions already have experience of online formation of companies. Some aspects of online company formation in Estonia, Latvia, Poland, and Ukraine are described, but not in as much detail as the characteristics of online company procedures in Lithuania, where most of the research methods, including the specially designed survey, were applied. Other jurisdictions are evaluated to the extent to which their provisions might be compared to Lithuanian law.

As part of the current research, a survey was carried out among representatives of large, middle-sized, and small law firms in Lithuania. Law firms were, *inter alia*, selected from the list of firms as provided by The Legal 500 in the jurisdiction’s overview.^19^ Among others, the following law firms were approached: CEE Attorneys, Ellex, Sorainen, WALLESS, ILaw/Lentax, Motieka & Audzevičius, SPS LEGAL, Glimstedt, Triniti Jurex, COBALT, Deloitte Legal, PWC Legal, TGS Baltic, Eversheds Saladzius, Sulija & Partners. Google Forms was used as a platform to send the questions to the corporate email addresses of law firms. The anonymous survey was conducted in February–March 2022. In total, 15 law firms and 25 company registration agencies were approached, and 13 answers were received to the survey: 4 respondents indicated that they represented large law firms and 9 respondents selected a small or medium-sized law firm as the group that they belonged to.

Additionally, an interview was carried out with a representative of the State Enterprise Centre of Registers regarding the implementation of the Digitalisation Directive, their experience in registering companies online and future work perspectives. Respective authorities in other jurisdictions, including the Centre of Registers and Information Systems of the Republic of Estonia, the Register of Enterprises of the Republic of Latvia, and the Ministry of Justice of the Republic of Poland, were also approached for statistical information about online registration of limited liability companies.

## Preparation and Content of the Digitalisation Directive

The Digitalisation Directive did not appear out of nowhere. The basis for the future proposal of the Digitalisation Directive draft was laid by expert reports. In 2016, the report of the Informal Company Law Expert Group (ICLEG) was issued.^20^ Among others, the ICLEG experts recommended changes in various areas including closer interaction of national systems and national business registers,^21^ online formation of companies in all EU Member States with model constituent documents for companies available online,^22^ and all filings relating to companies and their branches without physical presence.^23^ So, one of the ideas later embodied in the Digitalisation Directive and already pronounced by ICLEG experts regarding online formation of companies was the recommendation to make constituent documents for companies publicly available. That was a necessary step to launch remote registration of legal entities.

According to a 2016 research paper by Vanessa Knapp, a facilitative approach to digitalisation was suggested as it might be helpful. At the same time, the author admitted that the approach should be scrutinised.^24^ Without proper scrutiny, online company law procedures were viewed as carrying the threat of potential negative effect, including identity theft and corporate abuse.

One influential report was prepared in 2018 by Everis for the European Commission—DG Justice and Consumers.^25^ This study covered the company laws of 14 Member States, including the existing registration, filing, publication, and dissolution procedures therein.^26^ As a result of the study, several scenarios were developed for the implementation of digitalisation practices and a recommendation was issued to the EU to introduce online procedures for registration, filing, and voluntary dissolution where physical presence is not required.^27^

As a prelude to the adoption of the Digitalisation Directive, one may view the codification embodied in the Company Law Directive as well as the adoption of other acts of secondary legislation in the EU, including the Revised Shareholder Rights Directive,^28^ the Accounting Directive,^29^ the Directive for the interconnection of national registers,^30^ and the eIDAS Regulation, providing a regulatory environment for electronic transactions and identification.^31^ Thanks to the mentioned acts, one could witness the creation of an environment where online procedures in company law were a step ahead.

The Digitalisation Directive’s proposal was issued by the European Commission on 25 April 2018.^32^ In some provisions it traced back to the failed SUP Proposal, where the idea of online registration was embodied for single-member companies.^33^ Alessio Bartolacelli even called it ‘just a little more than the revival of the SUP proposal, with a broader extent and paying more attention to the role of notaries as of a company’s registration’.^34^ One of the main features of the proposal was claimed to be its heavy focus on the so-called ‘external digitalisation’,^35^ that is on relations between companies and states, including registration procedures, filing of information and documents, use of business registers, recognition of identification means and disqualification of directors. Based on the proposal’s analysis, Tina Jakupak and Željka Bregeš referred to the following main provisions of the proposal: registration of certain types of companies fully online; mandatory e-IDAS compliant identification for all EU citizens; online templates of constituent documents for certain types of companies; and the term for registration of a company—five working days after the submission.^36^ In other words, the proposal contained more Administrative Law than Company Law. The focus on purely private relations was reduced to a minimum. The final text of the Digitalisation Directive was signed on 20 June 2019.

The Digitalisation Directive provides for obligations of the EU Member States in three key areas: online formation of companies, online registration of branches, and online filing of information and documents. There are obligations that Member States have in all three mentioned areas, and certain more specific provisions regarding each area. On a more general level, every Member State shall designate an authority to deal with the mentioned online procedures.^37^ For all these types of online procedures, Member States shall ensure that concise and user-friendly information, provided free of charge and at least in a language broadly understood by the largest possible number of cross-border users, is made available on registration portals or websites that are accessible by means of the Single Digital Gateway.^38^ The Single Digital Gateway, established through the respective EU Regulation,^39^ should be able to facilitate access to online company law procedures through the common user interface. By using the Your Europe portal,^40^ one should be able to find and access the relevant national webpages describing online company law procedures in all the EU jurisdictions. The above would be in line with one of the EU Commission’s goals to provide the indicated information in a concise and user-friendly way as declared in a recital of the Digitalisation Directive.^41^

As far as online formation of companies is concerned,^42^ Member States must ensure that companies can be formed online without the need to appear in front of any authority or person. This obligation of Member States covers only legal persons indicated in Annex IIA, and for Lithuania this means that online formation of legal entities must work for private limited companies (UAB—*uždaroji akcinė bendrovė*). For other EU jurisdictions under investigation, the requirement to introduce online formation of companies similarly extends to private companies, in particular SIA (*sabiedrība ar ierobežotu atbildību*) in Latvia, OÜ (*Osaühing*) in Estonia, and sp. z o. o. (*spółka z ograniczoną odpowiedzialnością*) in Poland. Member States are free to introduce online formation procedures for other legal entities, but the minimum requirements of the Directive include various national business forms of private companies. It should be noted that when the Directive was still draft legislation an opinion was expressed that Member States should limit the type of companies formed online to those stipulated in Annex IIA.^43^ The idea behind such a recommendation was based on the fear that digitalisation would provoke fraud and, consequently, more public expenditures due to the creation of letterbox companies.^44^ This thesis again brings to mind the need for strong safeguards ensuing the proper identification of founders in the course of company formation, especially in cross-border cases.

Besides the general requirement to introduce online formation of companies, the Directive requires that there are detailed rules for online company law formation, including the possibility to have all documents or information submitted electronically (Art. 13g(2)). The detailed procedures as referred to in Article 13g(2) of the Directive include the necessary identification and verification procedures, procedures to verify the object of companies and the legality of company names, and procedures to verify the appointment of directors. The proper identification and verification of documents and company officers would have the aim of preventing any fraudulent behaviour directed at creating companies in breach of mandatory requirements. To implement and to enforce the mentioned requirements in the national laws, Member States would have to invest in human resources who would directly perform said verification procedures. It is also up to the Member States to do more and to provide more safeguards by providing additional checks involving notaries or other gatekeepers.^45^

One more important aspect of online formation of companies is related to the payment of share capital. Without the possibility of paying share capital online, the introduction of an online company procedure would be rendered largely superfluous because physical presence would still be required. That is why it is reasonable for companies formed online to have an option of online payment of share capital.^46^ The only exception to this rule that might be introduced by Member States is about contributions in kind: for these types of contributions, the physical presence of a founder makes sense as there would be a need to verify the substance and characteristics of the contribution to avoid its incorrect evaluation.

Although online formation of companies should be introduced, there is nevertheless an exception to this rule, stating that any authority or person authorised under national law to deal with online formation of companies may request the physical presence of the applicant.^47^ This can only be requested on a case-by-case basis if there is a risk of non-compliance with the rules of legal capacity of applicants. One example can be the risk of fraudulent behaviour based on crime records.^48^ This provision is in line with the more general requirement to ask applicants for physical presence only where it is justified by the reason of public interest, in preventing identity misuse or alteration.^49^ Some experts opine in favour of broader grounds to require physical presence to eliminate any potential risk of identity theft,^50^ but others view the current regulation as a preferred option.^51^ Indeed, if the grounds to require physical presence were too broad, such regulation could neutralise the whole idea of online company formation, which might then become an exception to paper format. Moreover, according to the information from the Centre of Registers of the Republic of Lithuania, during the period of more than 10 years for which online registration of companies has been possible in Lithuania, there have been no problems and no reported cases of fraudulent or falsified online registration—no-one disputed the establishment.^52^ There were only rare cases of ‘corporate theft’ of already existing companies a few years ago, when fictitious documents about the change of shareholders and directors were submitted to the Register and newly registered directors committed some criminal acts with those companies. This issue was solved by starting to require supporting documents for the transaction. However, the mentioned issue was not related to the online registration of companies.^53^

As for the online registration of branches, the Directive mandates that the registration of branches of companies created in other EU Member States can be performed fully online^54^ and for this purpose national rules should contain the respective identification and verification procedures.^55^ At the same time, there is no obligation for the EU Member States to lay down the conditions of registration for branches of companies formed outside the EU. Considering the risks associated with online company procedures, this way of regulation does not seem strange. A national authority registering a branch should be able to verify the capacity of the company officer(s)/body authorised to form a branch and the authority of a person representing the company. In a cross-border EU-non-EU context this might be more complicated due to limited access to foreign company registers, language issues, etc. Meanwhile, in the EU, since June 2017 business registers have been interconnected following the creation of the Business Registers Interconnection System (BRIS).^56^ The Digitalisation Directive requires that this system is used for the purposes of information verification and information exchange in case of registration of company branches.^57^ The Member State where the company is formed and the Member State where a branch is established are expected to exchange information. In addition, upon receipt of documents and information regarding closure of a branch in a Member State, when a branch is closed, the company shall inform the Member State where the company is established.^58^ Despite the digital transformations happening in the EU and in its Member States, so far in many cases the exchange of data has been carried out through the exchange of paperwork, but it is expected that the implementation of the Digitalisation Directive should streamline this process.^59^

All of the mentioned provisions had to be implemented by 1 August 2021,^60^ unless Member States applied for a 1-year extension.^61^ Among the studied jurisdictions, Poland has filed for an extension of the implementation deadlines, and so for Poland the first deadline was in August 2022. In addition, certain provisions need to be implemented by 1 August 2023.^62^ Those are the provisions about disqualified directors,^63^ verification of documents filed electronically,^64^ and provisions requiring that all documents and data are stored in machine-readable and searchable format or as structured data.^65^

## Legislative Developments on Digitalisation of Company Law in Lithuania

One of the key provisions of the Digitalisation Directive, the obligation to ensure online formation of private companies,^66^ was fulfilled in Lithuania in 2009–2010.^67^ The amendments to the Law on Companies^68^ adopted on 17 July 2009 gave way to model articles of association subject to separate approval by the government or its authorised institutions according to Articles 4(5) and 7(8) of the Law.^69^ Changes that were made to the Law on Companies allowed companies to be registered without the need to turn to a notary in case model documents were used. Model documents were consequently approved in 2009 by the Order of the Minister of Economy of the Republic of Lithuania.^70^ The option to form companies online became available on 3 November 2010, when the information system started functioning.^71^ At first it was only possible to form companies with one founder online, but in 2010 the Ministry of Economy approved a new model act where the possibility to have several founders in a private limited company was established.^72^

The introduction of model articles of association in Lithuanian law did not cancel the role of notaries in registering legal entities. It was still possible to turn to a notary for the certification of articles of association, for instance, when the model articles of association were insufficient so that more detailed or flexible provisions had to be inserted. At the same time, the adoption of model articles of association gave a new option to potential founders—to distantly create a company without directly meeting with any authority or person. The Lithuanian Chamber of Notaries expressed concern about the introduction of fully online registration of legal entities, but their opinion was not strong enough to overcome the government’s willingness to have online company formation without any intermediaries.^73^

The latest (2018) version of the model articles of association^74^ contains a set of standard provisions and a list of information that the founders must provide, including the company’s name, its business goals, its period of operation (unlimited or dated), the size of the authorised capital, number and types of shares, procedure of public announcements, and information about company bodies. By approving the template, the government implemented Article 13h of the updated Company Law Directive ahead of time.

Information about every legal entity in Lithuania can be found in the Register of Legal Entities, which functions in accordance with the Law on the Register of Legal Entities of the Republic of Lithuania.^75^ The Law provides for the interconnection of this register and registers of legal entities in other Member States of the EU.^76^ However, the latter act does not contain any information concerning electronic formation of companies in Lithuania. The mechanism for online formation of companies in Lithuania is in the respective Resolution of the Government of the Republic of Lithuania approving the Regulation on the Register of Legal Entities (hereinafter—the Register Resolution).^77^ The contents of the Register Resolution might be viewed as aimed at implementing Article 13g of the Company Law Directive, where the requirement to have detailed rules on company formation is in place. Currently, the founding documents may be submitted electronically for private companies, small partnerships (MB—*mažoji bendrija*), individual enterprises (IĮ—*individuali įmonė*), associations, public institutions (VšĮ—*viešoji įstaiga*), and charity and relief foundations (*labdaros ir paramos fondas*). In other words, in Lithuania not only private companies can be registered electronically. This electronic submission is carried out using the electronic signature, which until quite recently had to contain the personal identification code as a necessary attribute.^78^

On 2 June 2021, more amendments implementing the Digitalisation Directive in Lithuanian law followed in the new Resolution (hereinafter—Amending Resolution).^79^ The Amending Resolution was designed to take effect gradually: partly on 5 June 2021 and 1 January 2022, most of the paragraphs on 1 July 2022, and the rest on 30 July 2023. It is a complex document which inserts and substitutes paragraphs in the existing Register Resolution. The changes are meant to close gaps in the implementation of the Digitalisation Directive.

Firstly, the State Enterprise Centre of Registers is now authorised to provide data and documents to the European Commission as regards the Register of Legal Entities (implementation of Article 13c).^80^

Secondly, identification means issued by another Member State of the European Union in compliance with a sufficient level of trust according to the EU Regulations 1095/2010 and 910/2014 are now recognised—they should be published on the Registrar’s website (implementation of Article 13b of the updated Company Law Directive).^81^

Thirdly, pursuant to the Amending Resolution, information about the registration of legal entities and branches through electronic means shall be made available on the registration website in the official language and in any other language of the European Union that is understood by as many cross-border users as possible.^82^ Here, however, a difference between the text of the Digitalisation Directive and the Amending Resolution can be found. Article 13f of the updated Company Law Directive requires that the information should be concise, user-friendly, and at least in a language broadly understood by the largest possible number of cross-border users. As opposed to the Directive’s provisions, the implementing act does not mention the requirements of concise and user-friendly information. In addition, the Amending Resolution quite vaguely points at the language understood by the largest number of cross-border users. Considering that there is no need to regulate a specific language in the legislation, the implementation might be carried out by de facto actions of the government, i.e., by publishing concise and user-friendly information online in a foreign language. According to the information from the Centre of Registers of the Republic of Lithuania, a guide in English with illustrations was scheduled to launch for these purposes in July 2022.^83^ However, this was delayed because the corresponding functionality had to be implemented in the self-service system of the Centre of Registers. The system was launched only in February 2023.

Fourthly, while online formation of companies has been possible since 2010, the Amending Resolution has introduced online registration of branches^84^ (implementation of Article 28a of the Company Law Directive).

Fifthly, interconnection between registers during closure of branches (implementation of Article 28c of the Company Law Directive) is in place.^85^

Sixthly, disclosure of information, including a company’s name, code, website address, and other company information, is free of charge (implementation of Article 16a of the Company Law Directive).^86^

Seventhly, there is an interchange between registers of legal entities located in different EU Member States (implementation of Article 30a of the Company Law Directive).^87^

One of the changes the Amending Resolution has provided for is the new wording of Article 68 of the Register Resolution: now there is no mention of personal identification code as it no longer has to be encrypted in the qualified electronic signature used for online formation of private companies.^88^ According to the information from the Centre of Registers of the Republic of Lithuania, it was expected that recognition of qualified electronic signatures of all EU Member States would effectively start in early July 2022, but, again, processes have been delayed. The functionality was implemented on 18 November 2022, but some technical bugs were discovered, which were fixed, and this option became available to foreign founders in February 2023. This change should simplify the formation of companies online for foreigners coming to Lithuania and planning to obtain an electronic signature, because earlier the Centre of Registers accepted only those e-signature certificates that involved a Lithuanian personal code of the signatory. The qualified e-certificates were issued only to natural persons who have citizenship of the Republic of Lithuania or a permanent or temporary residence permit to stay in Lithuania.^89^ Also, thanks to STORK, a competitiveness and innovation framework programme co-funded by the EU, technical possibilities for recognition of qualified e-signature existed in respect of some EU Member States, such as Estonia, France, Greece, Italy, Luxembourg, The Netherlands, Portugal, Slovakia, Slovenia, Spain, and Sweden. STORK aimed at implementing an EU-wide interoperable system for recognition of eID and authentication in Member States.^90^

**Fig. 1:**
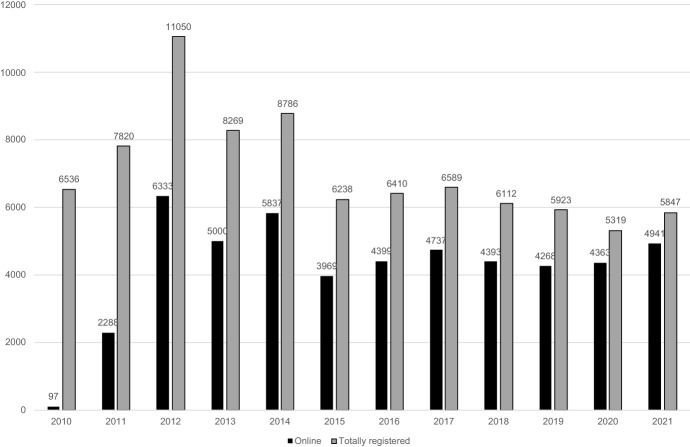
Statistics of registration of UAB in Lithuania from 2010 to 2021 (online and total). (Data received from the State Enterprise Centre of Registers of the Republic of Lithuania.)

Back in 2010, when the system of online registration started functioning in Lithuania, less than 2% (97 out of 6536) of private companies (UAB) were incorporated online. Since then, the quantity of legal entities created digitally has grown and online registration became available to more types of business organisations. In 2021, the situation changed: almost 85% (4941 out of 5847) of private companies (UAB) were established online (Fig. 1).

**Fig. 2:**
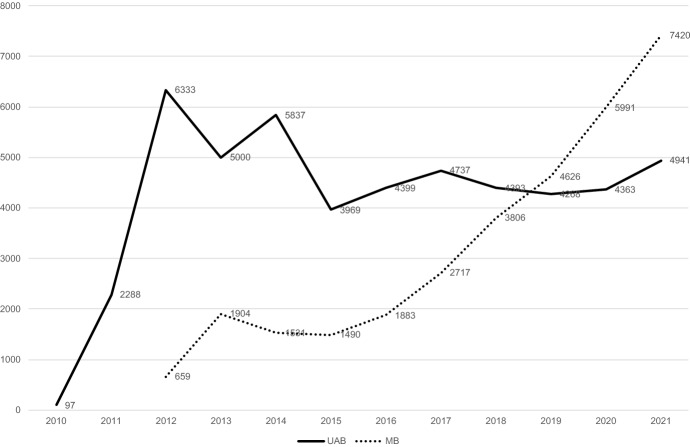
Statistics of online registration of UAB and MB in Lithuania from 2010 to 2021. (Data received from the State Enterprise Centre of Registers of the Republic of Lithuania.)

If we look at another legal form tailored for small and medium-sized businesses, small partnerships (MB), online registration is absolutely dominant. In 2021, almost 99% (7420 out of 7523) of small partnerships were formed online.^91^ Introduced in 2012,^92^ MBs enjoy certain benefits making them a popular form of business organisation (Fig. 2).^93^ As opposed to UABs, where the minimum share capital is EUR 2500 (from 1 May 2023 – EUR 1000), in MBs there is no minimum capital requirement and more flexible governance regulations. That is why MBs often serve as a simplified alternative to UABs.

## Comparative Aspects of Digitalisation: Estonia, Latvia, Poland, and Ukraine

Lithuania is not the only jurisdiction where online formation of companies was available before the Digitalisation Directive was adopted. It is necessary to consider how the introduction of online registration of companies progressed in other jurisdictions. A comparative overview of national laws on this matter would help in finding strengths and weaknesses of the national regulation in Lithuania and in suggesting improvements. For said purpose, Estonia, Latvia, Poland, and Ukraine are taken as jurisdictions with shared historic routes and similar legal systems and traditions. The selection of Ukraine as a jurisdiction beyond the European Union is additionally substantiated by its steps in digital transformation^94^ and European aspirations declared in the preamble of the Constitution of Ukraine.^95^

Estonia was among the first Member States to introduce online formation of companies in its legislation. It has been possible to establish private limited companies (OÜ (*Osaühing*)) online in Estonia since 2007.^96^ For that purpose one can use the special Company Registration Portal where templates are available.^97^ Both Estonian residents (owners of ID card, Mobile-ID, Smart-ID or Business Register users) and EU residents are able to register companies on the website. Foreigners able to register companies online in Estonia include, among others, users of the Lithuanian Mobile ID possessing personal identification codes, and owners of Latvian and Lithuanian Smart-IDs who are also in possession of personal identification codes.^98^

While in the case of private companies created online there is no need to turn to a lawyer because the founders insert the limited scope of information, including the business name, the registered office, the size of share capital, the percentage of votes required for the adoption of resolutions, the option pool, and the maximum number of members in a management board,^99^ for the registration of public limited companies (AS (*Aktsiaselts*)) one must turn to a public notary to certify the articles of association.

In 2018, the special group on modernisation of company law in Estonia presented a report with, *inter alia*, a suggestion to abolish the requirement of minimum share capital.^100^ This recommendation has been implemented recently. Previously, the minimum capital requirement of EUR 2500 was required for private companies in Estonia, although the legislation allowed for the creation of a limited liability company with delayed formation of share capital if it was less than EUR 25,000. Since 1 February 2023, the minimum capital requirement of EUR 2500 has been abolished and it can now be one cent per share in a private company.^101^ Where a share is paid for by a monetary contribution up to EUR 50,000, the fact that the contributions have been paid to the private limited company may be confirmed by the petition of the management board members. If the contribution is over EUR 50,000, a notice of a credit institution or payment institution is required.^102^

**Fig. 3:**
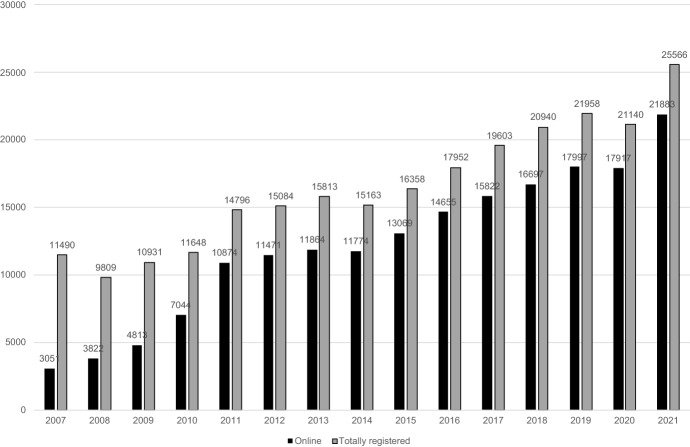
Statistics of online registration of OÜ in Estonia from 2007 to 2021 (online and total). (Data received from SIA ‘LURSOFT IT’.)

According to the information on registered undertakings in Estonia, in 2007, the first year of online formation of private companies, 26.55% of all incorporations of private companies that year (3051 out of 11,490) were carried out online.^103^ In 2021, 85.59% of private companies’ registrations (21,883 out of 25,566) were performed online (Fig. 3). These numbers clearly show that the digital formation of companies in Estonia has prevailed over paper-based incorporation.

In Latvia, it is allowed to form the following types of business organisations online: private limited company (SIA (*sabiedrība ar ierobežotu atbildību*)), general partnership (PS (*pilnsabiedrība*)), limited partnership (KS (*komandītsabiedrība*)), and, unlike other Baltic states, even a public limited company (AS (*akciju sabiedrība*)). The option to register companies online appeared in 2010: online incorporation and all changes in the register may be performed through a specially established e-portal: ‘Latvija’.^104^ All users must authenticate to use the e-service—this option is provided to the holders of electronic signatures recognised in Latvia (identity card eID, e-signature smart card or virtual e-signature).^105^ In addition, similarly to the Estonian approach, templates for the registration of companies are available online—forming a company would require inserting information about the name of the company, its share capital, and the number of members in the management board in the template.^106^

**Fig. 4:**
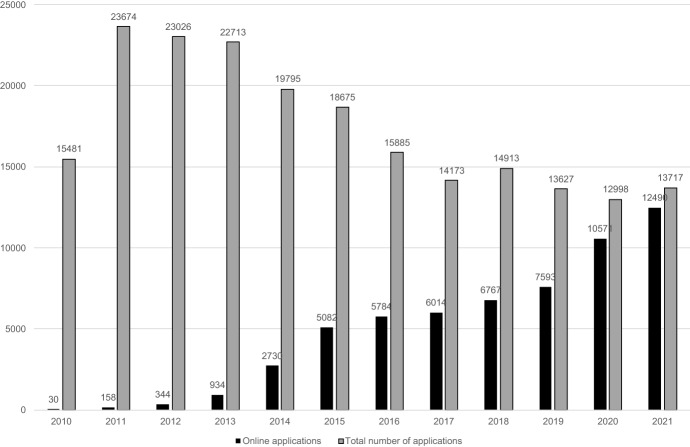
Statistics of applications for the registration of SIA, including Mazkapitāla SIA, in Latvia from 2010 to 2021 (online and total). (Data provided by the Register of Enterprises of the Republic of Latvia.)

For standard Latvian private companies, the minimum share capital is EUR 2800.^107^ In addition, since 2014 the government has allowed the establishment of low-capital SIAs, where share capital may constitute EUR 1,^108^ if the following criteria are met: not more than five founders (shareholders); all founders (shareholders) should be natural persons; board of directors should consist only of shareholders; and shareholders should own capital only in one low-capital SIA.^109^ In 2010, less than 0.2% (30 out of 15,481) of applications for the registration of limited liability companies were online, but over the years the situation changed dramatically: in 2021, 91% (12,490 out of 13,717) of applications were online (Fig. 4).^110^ Again, similarly to other Baltic states, paper applications for the registration of companies are now less popular than digital ones.

In Poland, it has been possible to form companies online since 2012.^111^ The Polish Code of Commercial Companies allows online formation of limited liability companies (sp. z o.o.) with a qualified electronic signature using the company template.^112^ Sp. z o. o. is the business form where the Digitalisation Directive requires online formation. The minimum share capital in a limited liability company is PLN 5000, which is more than EUR 1000.^113^ Besides limited liability companies, there are also other legal entities that might be established online, beyond what the Digitalisation Directive requests: simple joint stock companies (*prosta spółka akcyjna*), general partnerships (*spółka jawna*), and limited partnerships (*spółka komandytowa*).^114^ In contrast to limited liability companies, the minimum share capital in a simple joint stock company may even be PLN 1.^115^ The web portal which allows establishing companies in Poland is referred to as S24.^116^ Through this portal one may find the templates used for general partnerships, limited partnerships, and limited liability companies. Founders have to insert information about the name of the company, the size of share capital with contributions, the subject of the company’s activity, and the management’s term of office. In addition, founders have to select one of the options in the template regarding sale and pledge of shares (with or without the consent of the company), governance mechanism (with or without the supervisory board), advanced payment of dividends (yes or no), mode of representation of the company (in case of collegial body), and other options.

**Fig. 5:**
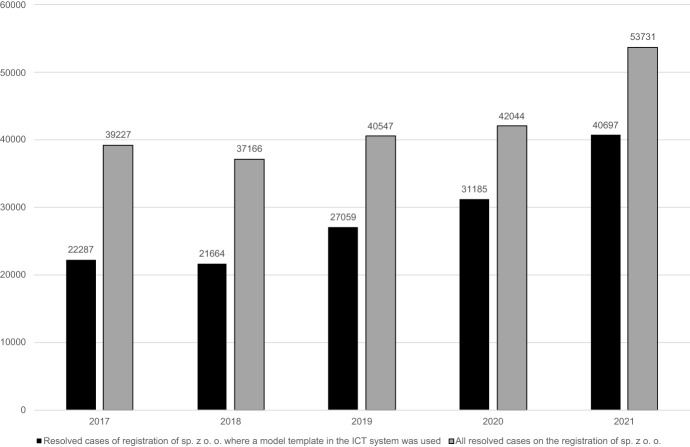
Statistics of resolves cases regarding the registration of sp. z o. o. in Poland from 2017 to 2021 (online and total). (Data provided by the Department for Strategy and European Funds of the Ministry of Justice of the Republic of Poland.)

When launched, the digital formation of companies in Poland was designed with the aim to facilitate and accelerate the establishment of those types of companies. At the same time, the Polish legislator, inspired by the example of Estonia, had to ensure certainty and safety of trading.^117^ During the period from January 2012 to August 2015, more than 31,000 limited liability companies were established using the template.^118^ As the Secretary of State in the Ministry of Justice, Michał Wójcik, noted in his reply to the member of the Polish Parliament from 30 June 2016, 49.95% of companies were registered at that moment in the so-called S24, meaning that every second company was registered in the ICT system using a template.^119^ According to the recent statistics, in 2021 online applications constituted almost 76% (40,697 out of 53,731) of all resolved cases of registration of limited liability companies (Fig. 5).^120^

At first, partnerships and companies could be formed using the electronic signature. Starting from 13 July 2017, due to the amendments in the legislation, the opportunity to use one of two identification options arose: a qualified electronic signature (a secure signature verified with a qualified certificate from a supplier recognised by the Ministry of Justice^121^), or a signature confirmed by the trusted ePUAP (Public Administration Services Platforms) profile.^122^ A signature obtained through the registration of a trusted profile in the ePUAP has been available to Polish nationals since 2017. Foreign nationals have also been able to obtain a trusted profile in case they have previously obtained a PESEL number with the Polish authorities. Several mentioned registration mechanisms have allowed for the provision of electronic identification of signatories, but nevertheless some gaps were inevitable. As has been rightfully observed by Marek Lesniak,^123^ even though the risks that existed before 13 July 2017 regarding the use of ‘ordinary’ certificates have been eliminated, there is still a risk of creation of false companies if a third party steals a signature or a PIN from a signatory (identity theft). As Lesniak emphasises, only physical appearance before a notary guarantees confirmation of identity.^124^ The approach where a notary always identifies a person shows the author’s adherence to the notarial system of entity registration. The essence of the latter system lies in the involvement of a notary in the verification of many transactions as an intermediary in relations between parties. This is where the Lithuanian legislator followed a different approach—to remove notaries from participating in the online company formation process altogether,^125^ and, as has been said, there have been no problems so far in Lithuania with fraudulent or false companies.

Ukraine is not obliged to implement the Digitalisation Directive as it is not yet an EU Member State. Nevertheless, it has already introduced regulation to form the basis of online formation of legal entities based on templates. The respective statute, which provided for the online creation of legal entities in Ukraine, was the Law of Ukraine adopted on 26 November 2015 and effective since 13 December 2015.^126^ The template for the registration of a limited liability company (TOV (*tovarystvo z obmezhenoiu vidpovidalnistiu*)) online in Ukraine was approved by the Cabinet of ministers of Ukraine in its Regulation dated 27 March 2019.^127^

**Fig. 6:**
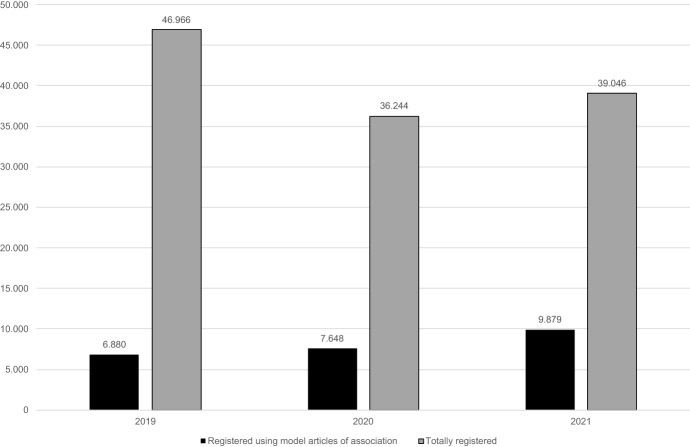
Statistics of registered limited liability companies (TOV) in Ukraine from 2019 to 2021 (using model articles of association and total). (Data provided by ‘YOUCONTROL’ LTD.)

In 2019, 14.65% of all registrations of limited liability companies in Ukraine (6880 out of 49,966) were carried out using the model articles of association. In 2021, one could see a slight change in numbers: 25.30% of private companies were established using the template (9879 out of 39,046) (Fig. 6).^128^

As the most popular legal entity in Ukraine,^129^ limited liability companies enjoy simplified conditions of registration and operation. There are no requirements as to the minimum share capital (share capital may amount to UAH 1), few necessary conditions to be included in the articles of association (company name; corporate bodies, their competence and decision-making procedure; company entrance and withdrawal from a company), and a simplified governance structure (there may be one director and a general meeting of shareholders).^130^ Said features make the mentioned form of business organisation an attractive one.

To form a limited liability company online in Ukraine a person would need a qualified electronic signature which can be obtained through a qualified provider of electronic trust services. One of the popular ways to obtain a qualified electronic signature is to receive it from Privat bank.^131^ A qualified e-signature obtained this way is valid for a certain period of time (usually a year), and is free of charge. After the expiration of the e-signature, a new one may be ordered. There is also a way to obtain an electronic signature on a separate material device. The legal basis for the mentioned procedures can be found in the Law of Ukraine ‘On Electronic Trust Services’.^132^ Every user of electronic trust services must be identified according to their name, surname and tax identification number or passport (in case the user due to religious beliefs does not want to obtain a tax number).^133^ Respectively, this means that a foreigner willing to obtain a qualified e-signature in Ukraine for the purpose of online company formation would have to first obtain their tax number in local tax authorities personally or through an authorised representative.

Using the e-signature, one may easily register a limited liability company through the Diia web portal.^134^ In addition, the Ministry of Justice of Ukraine offers online registration of non-governmental organisations.^135^ On the Diia portal, it is also possible to carry out authorisation through Bank ID and a signature created with Diia-pidpys. These online procedures were temporarily suspended due to the war in March 2022—only paper format was allowed.^136^

A feature of online formation of companies in Ukraine is that there are default provisions in the model articles of association in a limited liability company that may be subject to change. For instance, the default provision is that every company founder must make a contribution within 6 months after the state registration of a company, while other options include: 1 month, 3 months, and 1 year after registration. Other default provisions include, among others: additional term to pay debt—a time within 30 days established by the executive body in a company; additional contribution of a shareholder—proportionately to the share in charter capital of a company; time for third parties to make contributions in the amount of difference between the increased amount of charter capital and the amount of actual contributions—after every shareholder exercises one’s pre-emption right or refuses to exercise such a pre-emption right if the shareholders’ decision to attract new contributions stipulates so; and time for shareholders to attract additional contributions to a company—within the term established by the meeting of shareholders, but not more than a year after the decision to attract additional contributions. In general, there are more than 30 default provisions in the model charter of a limited liability company in Ukraine where other options may be chosen. This approach is similar to the one used in Poland, where founders also have several options when using the template of the articles of association of a limited liability company. The difference lies in the fact that in Ukraine there are more optional rules in the template.

The study of laws applicable to the registration of companies in Estonia, Latvia, Poland, and Ukraine shows that there are both differences and similarities in the online registration of legal entities. In all these jurisdictions, limited liability companies may be registered online. In Poland, besides the limited liability company (sp. z o. o.), there is the so-called simple joint stock company, which may also be registered online and by its features might be more attractive to investors than a limited liability company, as its minimum share capital is PLN 1, and it has a flexible governance structure. In Estonia, since 1 February 2023, legislation allows for the creation of a limited liability company with EUR 0.01 share capital, while in Latvia it is possible to register a limited liability company with EUR 1 capital under certain conditions outlined above. In Ukraine, a limited liability company may be established with UAH 1 share capital. So, for Estonia, Latvia and Ukraine, a limited liability company is still the simplest form of business organisation.

In general, out of those countries, Estonia has the longest history of application of online registration of companies (since 2007). This jurisdiction has for some time clearly given a way to form companies online to foreigners residing in the EU. In other studied EU jurisdictions, qualified electronic signatures from foreign providers have gradually been recognised. In Poland, there is also one more way to sign the founding documents: through the creation of an ePUAP trusted profile, but this covers only persons having a PESEL number. Therefore, qualified electronic signatures are the main identification tool to form companies online.

One cannot help mentioning the difference in templates in the mentioned jurisdictions. Estonia and Latvia use a rather standard template which does not allow different options of regulation. Meanwhile, in Poland and in Ukraine, templates are designed in a way that the founders may choose among various options. More discussion will follow in Sect. 6, where the results of a study of Lithuanian law will be analysed and discussed in combination with the results of the comparative overview presented in this chapter.

## Results of the Survey Among Law Firms in Lithuania

### Introductory Remarks

As has already been mentioned, a special survey was carried out among the representatives of large, medium-sized, and small law firms in Lithuania. The survey, *inter alia*, included online formation of companies. This section of the article presents the results (data) of the survey, while in the next section the results are discussed in combination with the application of other research methods, from the perspective of suitability, effectiveness, and sufficiency of regulation of this area in Lithuania.^137^

It should primarily be stated that out of 13 surveyed representatives of law firms, ten registered legal entities online. Four respondents registered up to 10 legal entities online, five respondents between 10 and 50 legal entities, and one respondent more than 50 legal entities online. Seven respondents reported issues when registering legal entities.

The issues emphasised by respondents regarding online formation of companies may be grouped into the following categories: (a) issues with the means of registration; (b) issues with the template of a constituent document; and (c) other issues. In the forthcoming paragraphs we discuss every category of these issues in detail.

### Feedback About the Means of Online Registration

Issues with the means of online registration were pointed out by several respondents. It was reported that, in the past, foreigners were not able to form companies online because only Lithuanian residents (Lithuanian nationals or persons holding temporary/permanent residence permit) having an electronic signature issued by the State Centre of Registers (a USB key), by the Identity Documents Personalisation Centre under the Ministry of the Interior (a special ID card), or by mobile operators in Lithuania could register companies online. Now, the need for the broader usage of various online registration means still exists, but to a limited extent. E-banking, among others, could not be used to sign documents, despite the fact there were suggestions to recognise it as a tool. It was moreover reported that some national electronic signatures were not recognised, despite the convenience of using them.

### Feedback About the Template of a Constituent Document

Online formation of a company requires a template of a constituent document. As has already been noted, the Lithuanian government updated the template for a private limited company in 2018. There were several critical comments in the survey regarding the existing template.

First, some respondents noted that it is not allowed to make the template bilingual, e.g., in Lithuanian and in English. However, this problem has already been solved by the Lithuanian registrar.^138^

Secondly, the existing template does not allow some non-standard provisions. Among others, it is not acceptable to provide a proxy in the articles of association or to provide additional functions to the supervisory board or to the general meeting of shareholders. In general, respondents expressed their opinion in favour of a more detailed and flexible document with more options available to company founders.

Overall, despite some critical comments, most respondents found the template for a private limited company suitable.

### Other Comments About Online Formation of Companies

Some comments were made that agents should have had the technical possibility and functionality to register legal entities—that was one of the reasons for some respondents not to register legal entities online and to turn to a notary instead. These respondents pointed out the need to accept all documents e-signed by them as agents not to overburden founders to take any new actions.

One more aspect where the work of agents is complicated is the reservation of a name of a company in the register. Firstly, there are limitations about the use of foreign names. Secondly, as has been reported by respondents, it is problematic to transfer the reserved name of a company to another person. Some respondents even suggested that the reservation of a company name in advance should be removed.

There were also comments regarding the fact that several documents had to be signed separately with an electronic signature instead of signing one file with several documents. These purely technical aspects were mentioned several times by respondents.

One more aspect with online registration of legal entities by foreigners was noted: charter capital of a legal entity must be formed from a bank account opened in a Lithuanian bank. That means that a bank account should be opened prior to the company registration. This aspect is further discussed in the next section of this paper.

### Overview of the Survey Results

Despite the existing issues outlined by respondents, most respondents nevertheless viewed the legal regulation of online formation of legal entities in Lithuania sufficient (out of 13, 2 called it absolutely sufficient and 7 fairly sufficient) and effective (out of 13, 4 called it absolutely effective and 4 fairly effective), while the template was viewed as suitable (out of 13, 7 called it fairly suitable and 2 absolutely suitable). Similarly, most respondents viewed the regulation of online filing of documents and/or information sufficient (out of 13, 6 viewed it as fairly sufficient and 3 as absolutely sufficient) and effective (out of 13, 8 saw it as fairly effective and 5 as absolutely effective).

## Discussion of the Survey Results with Comparative Analysis

### Means of Online Registration

As was shown in the previous section, several respondents opined in favour of broader usage of e-signatures during online formation of companies, namely the introduction of e-banking.

There are some arguments to support said statement. One argument lies in the fact that only contract-based clients of mobile operators can use mobile signatures. Prepaid mobile services do not give the opportunity to obtain a mobile electronic signature. To have a contract (usually a long-term contract) with a mobile operator, an individual would first need a personal identification number. In other words, a foreigner willing to obtain a mobile e-signature should first obtain a personal identification number (citizenship of the Republic of Lithuania or a permanent or temporary residence permit to stay in Lithuania) and then go to one of the stores of a mobile operator to sign an agreement.

Although mobile e-signatures in Lithuania exist only for contract-based clients, this does not mean that prepaid mobile customers should also have access to electronic signatures. The reason for this is that prepaid customers do not undergo any identification or security checks whatsoever. To the contrary, bank clients who have e-banking are always identified and always undergo a security check with the verification of identification documents. This is why there are grounds to recognise electronic banking as one of the identification tools for the purpose of company formation.^139^ Latvian and Ukrainian experience of using Bank ID as an identification tool proves that this instrument is user-friendly and convenient.

The problem of nation-specific requirements for electronic signatures existed before in Lithuania: in many other countries of the EU similar challenges were noted,^140^ including Latvia and Poland. Moreover, similar requirements could also be found outside the EU, namely in Ukraine, as outlined above. Since paragraph 68 of the Register Resolution, where the requirement to have a personal identification code included in the electronic signature is located, was amended by the Amending Resolution and the mention of the personal identification code was removed, a broader usage of various electronic signatures, including those issued abroad, was allowed. Since the Amending Resolution is aimed at implementing the Digitalisation Directive, it was in line with the latter^141^ in allowing electronic signatures where personal identification codes are not included.

### Template of a Constituent Document

One of the critical remarks of respondents concerned the language of the template (Lithuanian) and the fact that it was not previously possible to make it bilingual.

This problem is already in the past. According to information from the Centre of Registers, the opportunity to form bilingual (LT–EN) founding documents has already existed since December 2020. As was noted, when establishing companies online, bilingual (LT–EN) founding documents (articles of association, founding agreement/deed) as well as an application form can be selected.^142^

In terms of the official requirement, the Digitalisation Directive does not require templates to be in a language that is not official in a particular Member State; it only requires that the version in a language broadly understood by the largest possible number of cross-border users^143^ is on the website for information purposes. Nonetheless, considering the responses received from respondents of the survey and from the perspective of foreigners, the opportunity to make a bilingual constituent document (articles of association) is of much value.

The second comment from some respondents concerned the contents of the constituent document—they asked to make it more flexible, with more options available to founders. At the moment, the model articles of association of a limited liability company, as approved in 2018, has empty fields that the founders should fill out. Among others, founders should include information about company’s name, its business goals, the size of authorised capital, the number and types of shares, the procedure of public announcements, and company bodies. Based on the amount of required information, the regulations in Lithuania might be compared to those in Estonia and Latvia. Besides the more general information (company name, registered office and share capital), according to the Estonian regulations the percentage of votes for the adoption of resolutions, the option pool, and the maximum number of members in a management board should be stipulated. The Latvian template has even fewer spaces that should be filled out by founders: besides general information, the number of members in the management board should be indicated.

A different approach may be found in Poland and Ukraine, where various options are available to company founders. The Polish template has, *inter alia*, optional clauses about the sale and pledge of shares, governance mechanism, and the payment of dividends. The Ukrainian template has more than thirty default provisions, where founders may choose from a number of options.

Therefore, two approaches to composing templates are clearly visible in the studied jurisdictions: one suggests a template with many optional clauses, the other a template with standard provisions and limited information to be inserted at the registration stage. On the one hand, having optional clauses in the model articles of association provides more flexibility to founders—they may choose the clause which fits their needs. On the other hand, if the founders want more sophisticated articles of association, they can go to a notary. It may be argued that in a situation with many options of default provisions founders would be forced to hire lawyers to advise them how one option differs from the other, which is better to choose, etc. More legal expense is expected then. The incorporation process risks becoming even more costly if, instead of default provisions with many options, the model articles of association contain empty fields that the founders would have to fill out. Based on the above, introducing more default provisions for a template of a limited liability company is not a one-size-fits-all solution and should be studied more.

### Other Aspects of Online Registration of Companies

Some comments from law firms concerned the issues in the registration of companies by agents. Here, however, it should be emphasised that one of the cornerstones of the Digitalisation Directive is more rapid, easier, and more time- and cost-effective economic activity.^144^ The use of intermediaries makes any economic activity more expensive and cumbersome. That is why the problems faced by agents should be dealt with, as long as the implementation of the Directive is concerned, to the extent that it would simplify the direct registration process by founders, too.

One of the suggestions of respondents was to remove the reservation of a company name in advance as it was problematic to transfer the reserved name of a company to another person. Still, it seems that the reservation of a company name does not prove to be too problematic. The idea of this tool is to give time to founders to organise the company after they have agreed on the company’s name. Removing this mechanism might lead to some unnecessary risks with company formation when founders spend too much effort to create a company and at the end reveal that the company’s name has been taken by other founders.

Besides the requirement to reserve a name, there were a few complaints about the need to have a seat for a company. In 2018, there was a legislation proposal to allow legal entities to choose between a physical and a virtual registered office, i.e., a virtual account with the municipality and the address of the electronic delivery box in a special national information system.^145^ There are talks about a virtual seat for a company in legal doctrine as well,^146^ but this is not yet a reality across the board.

One more issue that has been emphasised by respondents was the issue of capital formation. In Lithuania, it is mandatory to pay authorised capital before registration, and for this purpose a bank account must be opened. Here respondents stressed that foreigners willing to form a company in Lithuania need to open bank accounts. Considering that the opening of a bank account may take an additional day, even when the registration itself lasts 10–15 min, such a requirement creates another barrier. In Estonia, this issue was dealt with by giving founders the opportunity to form capital in a limited liability company later if the amount of share capital does not exceed EUR 25,000. This right is restricted by the prohibition to increase or decrease capital until it is paid, as well as to make disbursements to shareholders for this time.

The Centre of Registers in Lithuania has embraced a different approach. Based on the information from the Centre of Registers, foreigners registering companies in Lithuania will be asked for a bank document confirming the fact of an opened account in an EU bank. For Lithuanian nationals, the approach will stay the same: they will need to open a bank account in Lithuania.^147^ This solution appears to simplify and to speed up the process of online company formation for foreigners.

### Concluding Remarks About the Survey Results

There were different problems that the respondents mentioned. Some of the outlined issues have already been tackled or should be resolved by the implementation of the Digitalisation Directive, namely the recognition of identification means issued in other EU Member States to empower foreigners and the publishing of constituent documents in a language broadly understood by most cross-border users. At the same time, some steps urged by respondents are beyond the requirements of the Digitalisation Directive. Those are both technical and legal measures. The recognition of e-banking as an identification tool would make online formation of companies more accessible to a broader range of people.

In addition, the respondents asked for legislation to go beyond the requirement of the Digitalisation Directive regarding templates and to provide more flexibility in their preparation—namely, to include provisions about proxies and to provide the opportunity to include more functions of corporate bodies. Overall, some provisions in the charter may be viewed as default, with options showing in the form of a drop-down list, but this approach also has its drawbacks outlined above and should be additionally investigated. As for purely technical issues, many respondents pointed at the apparently excessive need to have several documents signed with e-signatures instead of signing one single file.

In some directions where respondents expressed their remarks, the State Enterprise Centre of Registers of the Republic of Lithuania has already taken steps to make the process of registration more convenient. Among others, the Centre has worked to improve the language accessibility of the registration portal to foreigners and the bilingual (LT–EN) founding documents.

## Conclusions

One of the key provisions of the Digitalisation Directive is the obligation of EU Member States to ensure online formation of companies without intermediaries. This obligation was implemented by Estonia, Latvia, Lithuania, and Poland even before the Directive’s adoption (online formation of companies in Estonia has been available since 2007, in Latvia and Lithuania since 2010, and in Poland since 2012). The Lithuanian experience has, at the least, shown that the risk of fraudulent or falsified registration has not been established. A necessary feature of online company formation in Estonia, Latvia, Lithuania, Poland, and Ukraine is the presence of a model constituent document of a limited liability company (a template).

Besides the mentioned goals to ensure online company formation and to ensure the publication of a template of a private company, the Digitalisation Directive has other aims where further legislative actions were needed from the governments of the studied jurisdictions—the achieved results in online formation of companies were not enough to establish full implementation of the Digitalisation Directive. That is why the government of Lithuania adopted amendments to the legal acts to mostly take effect on 1 July 2022. Among others, the amendments provide for the recognition of identification tools, which means that electronic signatures issued in other EU Member States should be recognised. In addition, the State Enterprise Centre of Registers of the Republic of Lithuania has taken steps to change the registration portal’s interface, to have a guide in English and to simplify the process of registration by allowing foreigners not to have to open bank accounts in Lithuania as a prerequisite to incorporation.

Despite a considerably high level of implementation of the Digitalisation Directive in Lithuania, there are also issues in online formation of companies in Lithuania which were reported by respondents. To be specific, respondents mentioned the need to use e-banking for online company procedures and more flexibility in the signing of a template of a constituent document for a limited liability company. These suggestions essentially go beyond the effect of the Digitalisation Directive but are aimed at the improvement of the existing regulation of company formation. Therefore, to make online company formation more convenient it is not enough to be confined to the implementation of the Digitalisation Directive—one should look more broadly and consider the needs of the business community and the issues lawyers have faced when registering legal entities.

## Footnotes

1 Godwin et al. (2021), at p 11.

2 In Godwin et al. (2021), the influence of technologies on directors’ liabilities was the research focus of the chapter by Pey Woan Lee and Susanna HS Leong, while the characteristics of shareholder empowerment in the digital era was investigated by Pearlie Koh.

3 Bankewitz et al. (2016).

4 Enriques and Zetzsche (2020), at p 51.

5 Ibid., at p 6.

6 See, e.g., Page (2009,) at pp 248–283.

7 Hickman and Petrin (2021), at p 619.

8 Ibid., at p 620.

9 Directive 2017/1132 of the European Parliament and of the Council of 14 June 2017 relating to certain aspects of company law, OJ L 169, 30.6.2017.

10 Council of the European Union (2019).

11 Directive 2019/1151 of the European Parliament and of the Council of 20 June 2019 amending Directive (EU) 2017/1132 as regards the use of digital tools and processes in company law, OJ L 186, 11.7.2019.

12 Möslein (2019).

13 Omlor (2018).

14 Teichmann (2019).

15 Ibid., at p 7.

16 European Commission (2018).

17 Information from the official website of the European Union. https://eur-lex.europa.eu/legal-content/EN/NIM/?uri=CELEX:32019L1151&qid=1653660498251. Accessed 27 May 2022. Other Member States that have not applied for an extension include Ireland, Greece, Italy, Malta, Austria, and Portugal.

18 The conclusion about the implementation deadline is based on the information available at the governmental portal. https://www.gov.pl/web/premier/projekt-ustawy-o-zmianie-ustawy-o-krajowym-rejestrze-sadowym-oraz-niektorych-innych-ustaw2. Accessed 27 May 2022. Other Member States that requested extension include Croatia, Czech Republic, France, Germany, and the Netherlands.

19 Legal Market Overview. Coverage by practice area—Lithuania. https://www.legal500.com/c/lithuania/. Accessed 28 March 2022.

20 ICLEG (2016).

21 Ibid., at p 16.

22 Ibid., at p 18.

23 Ibid., at pp 20–21.

24 Knapp (2016), at p 4.

25 Everis, Study on digitalisation of company law. Publications Office of the European Union, Luxembourg. https://op.europa.eu/en/publication-detail/-/publication/89f1db3d-f849-11e8-9982-01aa75ed71a1, 2018.

26 Ibid., at p 8.

27 Ibid., at p 88.

28 Directive 2017/828 of the European Parliament and of the Council of 17 May 2017 amending Directive 2007/36/EC as regards the encouragement of long-term shareholder engagement, OJ L 132, 20.5.2017.

29 Directive 2013/34/EU of the European Parliament and of the Council, of 26 June 2013, on the annual financial statements, consolidated financial statements and related reports of certain types of undertakings, amending Directive 2006/43/EC of the European Parliament and of the Council and repealing Council Directives 8/660/EEC and 83/349/EEC, OJ L 182, 29/6/2013.

30 Directive 2012/17/EU of the European Parliament and of the Council of 13 June 2012 amending Council Directive 89/666/EEC and Directives 2005/56/EC and 2009/101/EC of the European Parliament and of the Council as regards the interconnection of central, commercial and companies registers, OJ L 156, 16.6.2012.

31 Regulation (EU) No 910/2014 of the European Parliament and of the Council of 23 July 2014 on electronic identification and trust services for electronic transactions in the internal market and repealing Directive 1999/93/EC, OJ L 257, 28.8.2014.

32 Council of the European Union (2019).

33 European Commission (2014a).

34 Bartolacelli (2018), at p 64.

35 Ibid., at p 63.

36 Jakupak and Bregeš (2020), p 21.

37 Company Law Directive, Art. 13c.

38 Ibid., Art. 13f.

39 Regulation (EU) No. 2018/1724 of the European Parliament and of the Council of 2 October 2018 establishing a single digital gateway to provide access to information, to procedures and to assistance and problem-solving services and amending Regulation (EU) No. 1024/2012, OJ L 295, 21.11.2018.

40 https://europa.eu/youreurope/. Accessed 6 June 2022.

41 Digitalisation Directive, recital 14.

42 Company Law Directive, Art. 13g.

43 European Trade Union Confederation (2021), at p 14.

44 Ibid., at p 7.

45 Company Law Directive, Art. 13g(3).

46 Ibid., Art. 13g(6).

47 Ibid., Art. 13g(8).

48 Omlor (2018), at p 6.

49 Company Law Directive, Art. 13b(4).

50 European Trade Union Confederation (2021), at p 9.

51 Dalli (2018), at p 3.

52 Interview with Jolanta Kazlauskiene, Head of the Department of the Register of Legal Entities of the Personal Registers Management Service at the State Enterprise Centre of Registers of the Republic of Lithuania, 1 June 2022.

53 Ibid.

54 Company Law Directive, Art. 28a(1).

55 Ibid., Art. 28a(3), (4).

56 Commission Implementing Regulation (EU) 2015/884 of 8 June 2015 establishing technical specifications and procedures required for the system of interconnection of registers established by Directive 2009/101/EC of the European Parliament and of the Council C/2015/3639, OJ L 144, 10.6.2015.

57 Company Law Directive, Art. 28a(5), (7).

58 Ibid., Art. 28c.

59 *Supra* n. 52.

60 Digitalisation Directive, Art. 2(1).

61 Ibid., Art. 2(2).

62 Ibid., Art. 2(3).

63 Company Law Directive, Art. 13i.

64 Ibid., Art. 13j(2).

65 Ibid., Art. 16(6).

66 Ibid., Art. 13g.

67 See Bitė (2017), at p 108.

68 Lietuvos Respublikos Seimas (2009).

69 Lietuvos Respublikos Seimas (2000).

70 Minister of Economy of the Republic of Lithuania (2009), Order No. 4-599 Regarding the confirmation of a model incorporation act form for the private limited liability company, model articles of association of the private limited liability company and model filling recommendations for incorporation act form for the private limited liability company and model articles of association of the private limited liability company, 18/11/2009. TAR, 21/11/2009, N. 138-6083.

71 Notification of the State Enterprise Centre of Registers, 3/11/2010.

72 Minister of Economy of the Republic of Lithuania (2010) Order No. 4-917 Regarding the confirmation of a model incorporation agreement form for the private limited liability company, model articles of association of the private limited liability company and model filling recommendations for incorporation agreement form for the private limited liability company and model articles of association of the private limited liability company, 16/12/2010. TAR, 23/12/2010, N. 151-7735.

73 Bitė and Gumuliauskienė (2016), at p 132.

74 Minister of Economy of the Republic of Lithuania (2018) Order No. 4-345 Regarding the approval of the model incorporation agreement of private limited liability company, model memorandum of private limited liability company, and model articles of association of private limited liability company, 11/06/2018. TAR, 13/06/2018, N. 9786.

75 Lietuvos Respublikos Seimas (2001).

76 Ibid., Art. 4-1.

77 Government of the Republic of Lithuania (2003).

78 Ibid., para. 68.

79 Government of the Republic of Lithuania (2021) Ruling on Resolution of 12 November 2003 amendment 1407 ‘On Approval of the Register of Legal Entities’, 2 June 2021, No. 393. TAR, 2021-06-04, no. 12911.

80 Ibid., para. 1.4.

81 Ibid., para. 1.7.9.

82 Ibid., para. 1.7.28.

83 *Supra* n. 52.

84 Government of the Republic of Lithuania (2021) Ruling on Resolution of 12 November 2003 amendment 1407 “‘On Approval of the Register of Legal Entities”’, 2 June 2021, No. 393. TAR, 2021-06-04, no. 12911, para. 1.7.24.

85 Ibid., para. 209-2.

86 Ibid., para. 251-1.

87 Ibid., paras. 1.7.38-1.7.41.

88 Ibid., para. 1.7.26.

89 See Bitė and Gumuliauskienė (2016), at p 135, Bitė (2017), at p 110.

90 See: https://joinup.ec.europa.eu/collection/secure-identity-across-borders-linked-stork. Accessed 22 June 2022.

91 Information received from the State Enterprise Centre of Registers of the Republic of Lithuania.

92 Lietuvos Respublikos Seimas (2012).

93 See Bitė and Gumuliauskienė (2015), at pp 359–360.

94 One of the goals of the Ministry of Digital Transformation of Ukraine as declared on its website (https://thedigital.gov.ua/ministry) is to provide 100% of public services to citizens and businesses online by 2024.

95 Verkhovna Rada of Ukraine (1996).

96 Riigikogu (2006).

97 https://ariregister.rik.ee/eng. Accessed 4 June 2022. Templates of model documents are also available at https://startupestonia.ee/resources. Accessed 4 June 2022.

98 https://ariregister.rik.ee/eng/application/start. Accessed 4 June 2022.

99 Riigikogu (1995), Art. 139-1.

100 Käerdi et al. (2018), at p 129.

101 Riigikogu (1995), Arts. 136 and 148.

102 Ibid., Arts. 144(2^1^) and 144(3).

103 Data received from SIA ‘LURSOFT IT’.

104 https://latvija.lv/Epakalpojumi/EP119/Apraksts. Accessed 4 June 2022.

105 https://latvija.lv/en/DzivesSituacijas/2250. Accessed 4 June 2022.

106 For instance, the template for a private limited company (SIA) is available here: https://www.ur.gov.lv/lv/registre/uznemumu-vai-komersantu/sia/dibinasana/registracija-uznemumu-registra/statuti/. Accessed 4 June 2022.

107 https://www.ur.gov.lv/en/register/company-or-merchant/limited-liability-company-sia/. Accessed 4 June 2022.

108 https://www.ur.gov.lv/en/register/company-or-merchant/llc-with-no-min-capital-requirement/. Accessed 4 June 2022.

109 Saeima (2000).

110 Information provided by the Register of Enterprises of the Republic of Latvia.

111 ICLEG (2016), p 75.

112 Sejm Rzeczypospolitej Polskiej, Art. 157-1. Kodeks spółek handlowych. Law of 15 September 2000. Dz. U. 2000, 94 (1037). http://isap.sejm.gov.pl/isap.nsf/download.xsp/WDU20000941037/U/D20001037Lj.pdf. Accessed 8 April 2022.

113 Ibid., Art. 154(1).

114 Ibid., Arts. 300-7, 23-1, 106-1.

115 Ibid., Art. 300-3.

116 https://ekrs.ms.gov.pl/s24. Accessed 6 June 2022.

117 Lesniak (2017), at pp 106, 232.

118 Ibid., at p 112.

119 Answer to interpellation No. 3468 on the system of electronic registration of companies S24. Responsible: Michał Wójcik, Secretary of State in the Ministry of Justice. Warsaw, June 30, 2016. https://www.sejm.gov.pl/sejm8.nsf/InterpelacjaTresc.xsp?key=2BED85C0&view=null. Accessed 6 June 2022.

120 Information provided by the Department for Strategy and European Funds of the Ministry of Justice of the Republic of Poland.

121 List of certificates recommended by the Ministry of Justice, http://www.nccert.pl/. Accessed 6 June 2022. The list does not contain any foreign issuers of certificates.

122 Lesniak (2017), at p 130. The platform is available at: http://epuap.gov.pl/wps/portal. Accessed 6 June 2022.

123 It should be noted that Marek Lesniak has chaired the commission drafting a legislation response to the Directive in Poland since around mid-2020. Komisja ds. reformy nadzoru właścicielskiego wykorzysta nowe technologie w prawie handlowym. 24.07.2020. https://www.gov.pl/web/aktywa-panstwowe/komisja-ds-reformy-nadzoru-wlascicielskiego-wykorzysta-nowe-technologie-w-prawie-handlowym. Accessed 12 April 2022.

124 Lesniak (2017), at p 263.

125 Bitė and Gumuliauskienė (2016), at p 132.

126 Verkhovna Rada of Ukraine (2015) Law of Ukraine ‘On Amendments to the Law of Ukraine ‘On State Registration of Legal Entities and Natural Persons—Entrepreneurs’ and Certain Other Legislative Acts of Ukraine regarding the Decentralization of Competence regarding State Registration of Legal Entities, Natural Persons—Entrepreneurs and Non-Governmental Organisations’ No. 835-VIII dated 26 November 2015.

127 Cabinet of Ministers of Ukraine (2019).

128 Data provided by ‘YOUCONTROL’ LTD.

129 On 1 April 2022, there were 748,672 limited liability companies out of 1,443,030 officially registered legal entities in Ukraine, i.e., almost 52%, according to the State Service of Statistics of Ukraine: http://www.ukrstat.gov.ua/edrpoy/ukr/EDRPU_2022/ks_opfg/ks_opfg_0422_ue.xls. Accessed 6 June 2022.

130 Verkhovna Rada of Ukraine (2018) Law of Ukraine ‘On Limited Liability companies’ No. 2275-VIII dated 6 February 2018, Arts. 11(5), 12, 28, 39.

131 Receipt of the certificate. https://acsk.privatbank.ua/key. Accessed 15 April 2022.

132 Verkhovna Rada of Ukraine (2017) Law of Ukraine ‘On Electronic Trust Services’ No. 2155-VIII dated 5 October 2017.

133 Ibid., Art. 23(2).

134 Registration of LLC based on model template. https://diia.gov.ua/services/reyestraciya-tov-na-pidstavi-modelnogo-statutu. Accessed 15 April 2022.

135 Online house of justice. https://online.minjust.gov.ua/. Accessed 16 April 2022.

136 Cabinet of Ministers of Ukraine (2022).

137 It should be borne in mind that the survey was carried out before the taking of effect of the Amending Resolution. Therefore, all feedback received from the surveyed law firms was based on the experience received from the use of the Register Resolution in the previous version.

138 See in Sect. 6.2.

139 E-banking, for instance, is used in another area alongside mobile electronic signatures and other electronic signatures—on the medical portal https://www.esveikata.lt/.

140 European Commission (2014b), at p 27.

141 Company Law Directive, Art. 13b.

142 *Supra* n. 52.

143 Company Law Directive, Art. 13h(3).

144 Digitalisation Directive, recital 2.

145 Government of the Republic of Lithuania (2018).

146 Mikaloniene (2022), at p 5.

147 *Supra* n. 52.
